# Morphological Computation of Haptic Perception of a Controllable Stiffness Probe

**DOI:** 10.1371/journal.pone.0156982

**Published:** 2016-06-03

**Authors:** Nantachai Sornkarn, Prokar Dasgupta, Thrishantha Nanayakkara

**Affiliations:** 1 Centre for Robotics Research, Department of Informatics, King’s College London, London WC2R 2LS, United Kingdom; 2 MRC centre for Transplantation, DTIMB and NIHR BRC, King’s College London, Guys Hospital, London SE1 9RT, United Kingdom; Massachusetts Institute Of Technology, UNITED STATES

## Abstract

When people are asked to palpate a novel soft object to discern its physical properties such as texture, elasticity, and even non-homogeneity, they not only regulate probing behaviors, but also the co-contraction level of antagonistic muscles to control the mechanical impedance of fingers. It is suspected that such behavior tries to enhance haptic perception by regulating the function of mechanoreceptors at different depths of the fingertips and proprioceptive sensors such as tendon and spindle sensors located in muscles. In this paper, we designed and fabricated a novel two-degree of freedom variable stiffness indentation probe to investigate whether the regulation of internal stiffness, indentation, and probe sweeping velocity (PSV) variables affect the accuracy of the depth estimation of stiff inclusions in an artificial silicon phantom using information gain metrics. Our experimental results provide new insights into not only the biological phenomena of haptic perception but also new opportunities to design and control soft robotic probes.

## Introduction

Physical examination of soft objects to identify hidden mechanical features can be seen in a variety of areas like minimally invasive surgery, medical physical examination, security, quality assurance in food industry, entertainment, etc. Manual examination involves variation of both behavioral and internal impedance levels of the fingers [1, 2], because the interplay between motor control and internal mechanics (muscles and reflexes) [3, 4] play an important role in both action and perception [5]. The notion—*morphological computation*—in soft robotics and biological systems views the mechanical circuits in the embodiment as a computational resource for both perception and action. Since the mechanical impedance of the embodiment changes the functionality of those mechanical circuits, it is important to understand its role in regulating perception and action of soft robots. The concept of internal impedance control in dynamic systems was first laid out by Hogan in [6]. However, its applications have been largely limited to control of action than understanding its role in perception or action-perception coupling in biological systems. For instance, impedance control principles have been widely applied in robotics for metastable walking [7], tele-operated excavation [8], safe interaction with humans [9, 10], and to enhance stability of prosthetic devices [11]. They take advantage of passive body dynamics and the interaction with environment to achieve required goals without high-level cognitive processes [12].

**Fig 1:**
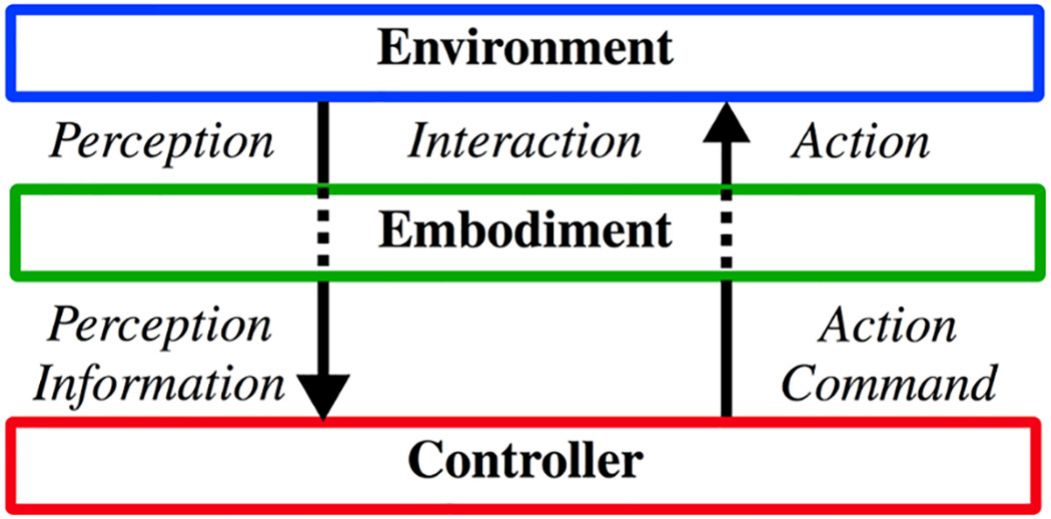
Embodiment mediates both perception and action during the interaction between an agent and the environment. The system interacts with the environment through its embodiment. The internal impedance required for accurate perception through its embodied sensor can differ from that required to take appropriate action. Likewise an action taken with appropriate impedance could affect the quality of perception of the environment.

Fig 1 illustrates how perception and action are coupled when they share a common embodiment. In biological systems, proprioceptive sensors such as spindle sensors (sense the amount and speed of muscle contraction) and tendon sensors (sense force) are located in the very muscles that are used to actuate joints. Therefore, controllers in the central nervous system perceive the environment depending on the actuation state of the muscles and muscle actuation in turn depends on perception [13]. Even in tactile perception [14], different types of mechanoreceptors are positioned at different depths in the dermis [15] to exploit different features of tissue dynamics. How this interplay among muscle actuation behavior, environment, and co-contraction of antagonistic muscles affect the accuracy of proprioception is not well quantified yet [16].

Recently, we have explored the role of internal impedance control for maximizing the information gain in robotic embodied perception [17]. The analytical results and experimental evidence have signified that a robotic manipulator with controllable internal impedance can maximize information gain (measured using transfer entropy) by searching for an optimal stiffness under a non-linear relationship between the entropy of sensory information and the impedance of physical embodiment of the robot. This paper addresses the question as to whether a robotic probe with variable impedance, indentation, and probe sweeping velocity (PSV) can achieve a better estimation accuracy of a given environmental condition (i.e. depth of a nodule embedded inside a silicon phantom).

In order to address this question, we designed and fabricated a new soft robotic probe with a controllable stiffness Mckibben type joint. The setup for probing experiments on a soft silicon phantom to estimate the depth of an embedded nodule is described in the following section. Then the dynamic simulations were carried out to explore the individual and collective role of internal impedance, indentation level, and PSV in the measured torque response. In the experimental section, we discuss experimental results derived from 5625 probing trials, where we explain our main contributions—1) Evidence to show that the internal stiffness of the soft probe plays a statistically significant role in the accuracy of nodule’s depth estimation, 2) A Bayesian learning framework to combine internal stiffness, indentation level, and PSV that maximizes the information gain, and 3) Experimental results to show that proposed algorithm can achieve on average 99% and 96% accuracy in estimating the nodule’s depth in both active and passive perception respectively.

## Design and Analytical Model of Variable Stiffness Probe

### Design

**Fig 2:**
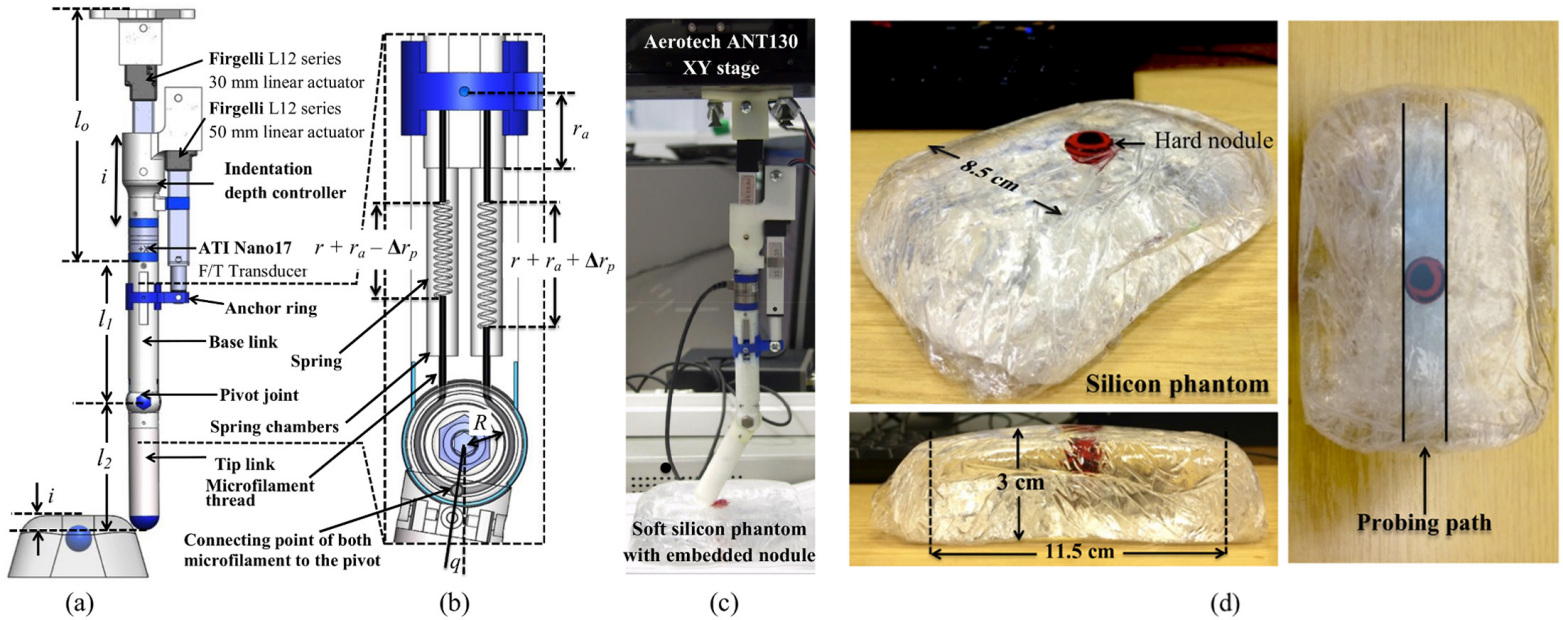
Three dimensional design of the robotic probe and photos of experimental setup and sample soft silicon phantom. (a) Shaded view of two-link probe’s design. (b) Two springs located inside the spring chambers are attached with the anchor ring and the pivot joint through a microfilament thread. (Note that the springs shown here are for illustrative purpose only). The stiffness of the joint can be represented by the distance of *r*_*a*_. (c) Photo of the complete experimental platform’s design comprising of the variable stiffness probe mounted on XY-stage. (d) A soft silicon phantom fabricated using soft clear silicon elastomer gel with a spherical plastic bead of size 15mm diameter embedded inside at different depths.

The overall design of the probe used in this experiment is depicted in Fig 2(a). It is composed of two rigid links—tip link with length, *l*_1_ = 80 mm, and base link with length, *l*_2_ = 70 mm—made from ABS plastics. The joint connecting between these two links are coupled with a variable stiffness mechanism comprising of two identical linear ENTEX No.3552 stock springs (0.24 N/mm) from Advanex Europe Ltd, to represent how antagonistic muscles control the stiffness of biological fingers. Both springs are situated in spring chambers inside the base link and suspended between the pivot joint (at the connecting location, at which the relative angle to the vertical axis of the tip link is zero) and the movable anchor ring through a micro-filament thread (see Fig 2(b)). The stiffness of the joint can be regulated by changing the position of the anchor ring, *r*_*a*_, which is controlled by a 50 mm-linear actuator L12-50-210-06-I from Firgelli Technologies Inc. An ATI Nano17 F/T transducer is mounted at the top-end of the base link to measure the torque during the interaction with soft silicon phantom. This represents how the tendon sensor is located at the top end of a natural muscle.

The probe’s indentation level, *i* is controlled by a 30mm-linear actuator (L12-30-100-06-I from Firgelli Technologies Inc) mounted at the top of the base link. The total length of this mechanism when at rest is denoted by *l*_*o*_ and has the initial value of 140mm. Hence the total rest length of this probe when the angle of pivot joint, *q* = 0, is 290mm. The probe structure is mounted on a flipped ANT130 XY-stage with built-in speed regulator from Aerotech Inc. as shown in Fig 2(c), which allows the planar movement in x- and y-direction at a constant speed. During the interaction with environment, e.g. palpation, the torque around the x-axis is measured at the end of the base link of the probe using an ATI Nano17 Force/Torque (F/T) transducer.

Here, we used soft silicon phantoms with an embedded hard nodule as the samples for the haptic perception experiments. Silicon phantom is made from a soft clear silicon elastomer gel RTV27905 from Techsil. The given chemical substances (Part A and B) were mixed in 1:1 ratio according to specification to fabricate the silicon phantom. According to the studies in human biology, the typical tumor in soft tissue has roughly spherical shape [18] and at T1 stage can vary in size between 0.5 to 20mm [19]. Hence a spherical plastic bead with a diameter of 15mm was embedded inside each phantom at different depths (see Fig 2(d)). In this experiment, three silicon phantoms were used as samples where the nodules were embedded at the depth of 2, 4, and 8mm from the top surface of the phantom to the top of a nodule.

### Variable Joint Stiffness

The exploded view of the variable stiffness joint is shown in Fig 2(b). At rest (the angular displacement, *q* = 0 and the position of anchor ring, *r*_*a*_ = 0), the rest length of both springs are equal and denoted by *r*. Both springs can be extended from their rest by changing the position of the anchor ring, *r*_*a*_. The change of the angular displacement of the joint, *q*, due to the passive interaction with the environment, depending on the direction, causes both springs to be compressed and extended by Δ*r*_*p*_, where Δ*r*_*p*_ = *qR*. *R* is the radius of the pivot joint at which the microfilament is attached to. Hence the change of the length of the springs can be computed as following:

$$\Delta r_{1}=r_{a}-qR$$ (1)

$$\Delta r_{2}=r_{a}+qR.$$ (2)

Since both springs are identical, we can compute the force contributed from each spring to the probe’s joint as follows:

$${\vec{f}}_{s1,2}=\Delta r_{1,2}k_{s}.$$ (3)

Hence, the torque generated from both springs due to the change of joint’s angular displacement and the position of the anchor ring is:

$$\tau_{s1,2}={\vec{f}}_{s1,2}\times R={\vec{f}}_{s1,2⊥}R,$$ (4)

where ${\vec{f}}_{s1,2⊥}$ is the force produced from each spring perpendicular to the rotational axis.

$${\vec{f}}_{s1,2⊥}=f_{s1,2}\sin\left(q\right).$$ (5)

Therefore, the total torque developed due to both springs can be computed from Eqs (3) to (5) as follows:

$$\begin{array}{ccc} \tau_{s} & = & \tau_{s1}+\tau_{s2}\\ & = & Rk_{s}\sin\left(q\right)\left(\Delta r_{1}+\Delta r_{2}\right) \end{array}$$ (6)

and the stiffness at the joint, *K*_*s*_, is the derivatives of torque produced with respect to the angular displacement of the pivot joint, *q*, from Eq (6)

$$K_{s}=\frac{\partial \tau_{s}}{\partial q}=2r_{a}Rk_{s}\cos\left(q\right).$$ (7)

**Fig 3:**
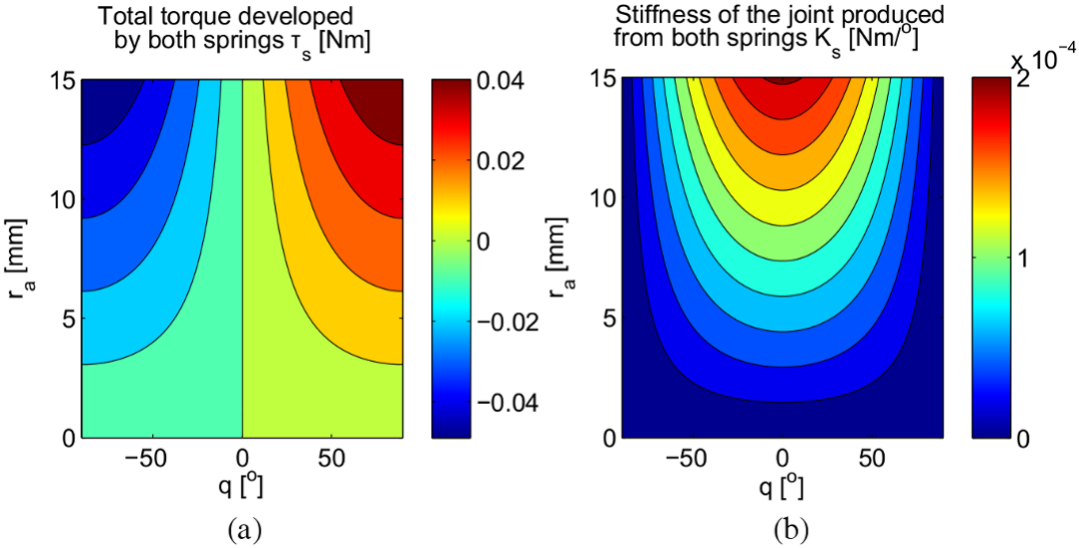
Simulated joint torque and stiffness. Torque (a) and the stiffness (b) produced at the pivot joint due to the changes in the displacement of the anchor ring, *r*_*a*_, and the angular displacement of the joint, *q*.

From Eqs (6) and (7), we can simulate the torque, *τ*_*s*_, as well as the resulting joint stiffness, *K*_*s*_, as function of *q* and *r*_*a*_. The simulation results shown in Fig 3 are generated from the following parameters: *r*_*a*_ = [0…15]mm, *R* = 6.8mm, *q* = [−90…90]°, and *k*_*s*_ = 0.24N/mm.

The resulting joint torque due to the changes of both parameters, *q* and *r*_*a*_, is shown in Fig 3(a). Here we can see that the extension of both springs by increasing *r*_*a*_ results in a change of the landscape of the relationship between *τ*_*s*_ and *q*. Taking derivatives of the simulated joint torque with respect to the angular displacement of the pivot joint results in the stiffness profile of the joint shown in Fig 3(b). The stiffness of the joint becomes almost linear as the anchor ring approaches its origin. Since *r*_*a*_ can be controlled through the linear actuator, for the rest of this paper, we represent the joint’s stiffness level in term of the position of the anchor ring, *r*_*a*_ mm.

### System’s Dynamics

**Fig 4:**
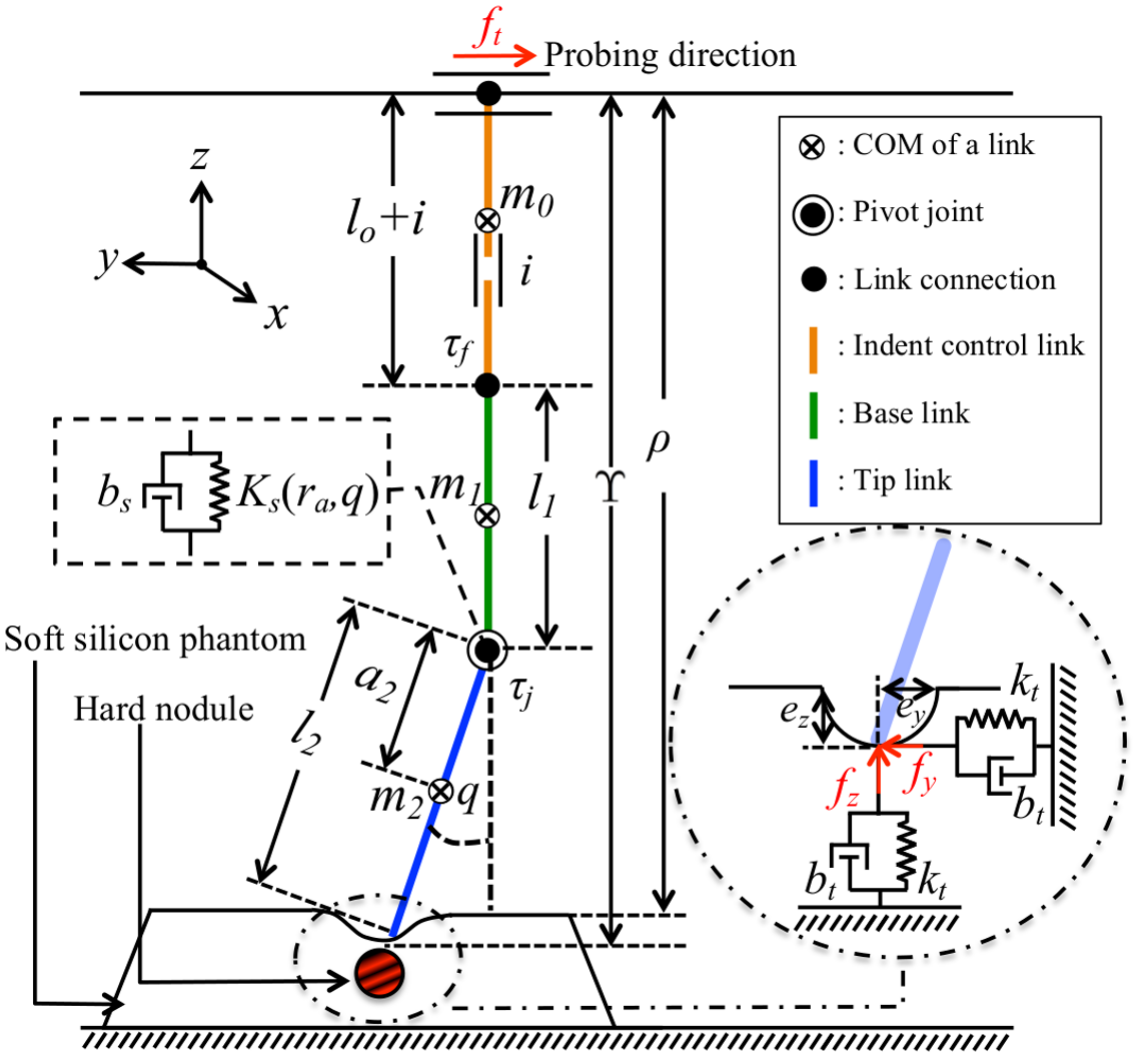
Schematic view of the probe interacting with simulated soft silicon phantom. The probe consists of two rigid manipulator links with a variable stiffness joint. In the experiment, *τ*_*f*_ is measured at the end of base link of the probe using ATI Nano17. The nodule embedded inside the phantom is shown in red-black spherical ball. The physical properties of phantom is described using spring-damper system. The variables used in the system is described in Table 1.

The description of variables used in the system depicted in Fig 4 is shown in Table 1. The joint coupled between the tip and base link comprises of a variable stiffness element, which is represented by a variable spring-damper system. According to the derivation shown in Eq (7), the variable joint’s stiffness, *K*_*s*_(*r*_*a*_, *q*), is therefore a function of the position of anchor ring, *r*_*a*_, and the angular displacement of the pivot joint, *q*.

**Table 1:** System’s variables.

System	Variables	Descriptions
**Probe**	*τ*_*f*_	Torque measured at the end of base link
	*τ*_*j*_	Torque at the pivot joint
	*q*	Angular displacement of pivot joint
	*l*_*o*_	Length of the Indentation control link
	*l*_1_	Total length of Base link
	*l*_2_	Total length of Tip link
	*a*_2_	Distance to center of mass of tip link
	*m*_0,1,2_	Mass of Indent control, Base, and Tip link
	*i*	Indentation
	*g*	Gravitational Constant [9.81 ms^−2^]
	*b*_*s*_	Damping coefficient of variable stiffness joint
	*K*_*s*_(*r*_*a*_, *q*)	Joint’s variable stiffness rating
	*ρ*	Distance from XY table to phantom’s surface
	*e*_*y*,*z*_	Phantom’s deformation in y- and z-direction
	*ϒ*	*ρ* + *e*
	*f*_*t*_	Translational force in probing direction
**Phantom**	*b*_*t*_	Damping coefficient of silicon phantom
	*k*_*t*_	Stiffness of silicon phantom
	*f*_*y*_	Restoring force from phantom in y-direction
	*f*_*z*_	Restoring force from phantom in z-direction

#### Equation of Motion

The interaction dynamics of the system can be derived as follows:

$$m_{2}a_{2}^{2}\ddot{q}+m_{2}ga_{2}s+K_{s}q+b_{s}\dot{q}=\tau_{j},$$ (8)

where *τ*_*j*_ is the torque at pivot joint. The torque, *τ* = (*τ*_*f*_, *τ*_*j*_)^T^ can be resolved from the force components in y- and z-direction, **f** = (F_*y*_, F_*z*_)^⊤^, at the probe’s tip during the interaction with soft silicon phantom. *τ*_*f*_ denotes the measured torque at the end of base link. The descriptions of the variables used here are explained in Table 1. Note that in this paper, the trigonometric functions are abbreviated as follow: *s* = sin(*q*) and *c* = cos(*q*).

In order to simplify the dynamic equations of the system, the restoring force of the silicon phantom on the probe during the interaction can be modeled using a linear spring-damper system, where the stiffness of the silicon depending on different depth levels of a hard nodule embedded in the sample phantom can be represented by varying the system’s spring constant.

Assume that:

1. At rest (no contact between probe and sample phantom) the probe has length of *L* = *l*_*o*_ + *l*_1_ + *l*_2_ + *i*.
2. The base of the probe is fixed directly above the sample phantom at distance *ρ*.
3. The probe has stiffness *K*_*s*_ as a function of *r*_*a*_ and *q*.
4. The soft silicon phantom has uncertain stiffness *k*_*t*_ with Gaussian distribution.
5. The restoring force from the soft silicon phantom is in both y and z-direction.
6. The friction between the tip and soft phantom’s surface is negligible.
7. The deformation of soft silicon phantom has a uniform curvature [20].

When the probe comes in contact with the sample phantom, both the probe and the phantom deform according to their relative stiffness as shown in Fig 4.

We denote the depth of phantom sample deformation in y- and z-directions by *e*_*y*_ and *e*_*z*_ respectively. The probe length (compressed prismatically), ϒ, can be expressed as a function of *q* as

$$\Upsilon =l_{o}+i+l_{1}+l_{2}c.$$ (9)

Since the base of the probe is fixed, the constraint

$$|e_{z}|=\Upsilon -\rho$$ (10)

is maintained. Furthermore, we assume a uniform curvature deformation (the magnitude of deformation in both y- and z-directions are equal) of the soft silicon phantom. Therefore:

$$|e_{y}\left|=\right|e_{z}|.$$ (11)

By substituting Eq (9) in Eq (10), we obtain:

$$|e_{y}\left|=\right|e_{z}|=\left(l_{o}+i+l_{1}+l_{2}c\right)-\rho$$ (12)

$$\dot{e_{y}}=\dot{q}l_{2}c$$ (13)

$$\dot{e_{z}}=-\dot{q}l_{2}s.$$ (14)

The restoring force from the soft silicon phantom on the probe in both directions can be modeled as a spring-damper system as follows:

$$f_{y}=k_{t}e_{y}+b_{t}\dot{e_{y}}$$ (15)

$$f_{z}=k_{t}e_{z}+b_{t}\dot{e_{z}}.$$ (16)

Substituting Eqs (12)–(14) in Eqs (15) and (16), we obtain:

$$f_{y}=k_{t}\left(l_{o}+i+l_{1}+l_{2}c-\rho \right)+\dot{q}l_{2}b_{t}c$$ (17)

$$f_{z}=k_{t}\left(l_{o}+i+l_{1}+l_{2}c-\rho \right)-\dot{q}l_{2}b_{t}s.$$ (18)

Therefore the force component due to the interaction with soft silicon phantom at the tip is **f** = (F_*y*_, F_*z*_)^⊤^, where

$$F_{y}=f_{y}-f_{t}$$

$$F_{y}=f_{y}-\left(m_{0}+m_{1}+m_{2}\right)\ddot{y}\;\text{and}$$ (19)

$$F_{z}=f_{z}.$$ (20)

*F*_*y*_ and *F*_*z*_ are net force in y- and z-direction. *f*_*t*_ is the translational force in probing direction. $\ddot{y}$ denotes the translational acceleration. Note that *f*_*y*_ and *f*_*z*_ contain variables, dependent on the terms indicated, since it is a function of the random variable *k*_*t*_. The terms $r_{a},q,\dot{q},$ and *i*, can be thought of as parameters to the distribution. Adjusting any of these may have an effect on the information in samples of **f**.

#### Torque measurement model

In the design of the probe shown in Fig 2(a), torque, *τ*_*f*_, is measured at the end of the base link. The Jacobian matrix, **J** of the system can therefore be expressed as:

$$J=\left(\begin{array}{cc} l_{1}+l_{2}c & l_{2}c\\ -l_{2}s & -l_{2}s \end{array}\right).$$ (21)

We get the equations of torque resulting from the interaction with the soft silicon phantom as follows:

$$\begin{array}{ccc} \tau & = & J^{⊤}\text{f}\\ \tau =\left(\begin{array}{c} \tau_{f}\\ \tau_{j} \end{array}\right) & = & \left(\begin{array}{cc} l_{1}+l_{2}c & -l_{2}s\\ l_{2}c & -l_{2}s \end{array}\right)\left(\begin{array}{c} F_{y}\\ F_{z} \end{array}\right). \end{array}$$ (22)

Hence the torque measured by the ATI Nano17 transducer mounted at the end of the base link can be derived as:

$$\tau_{f}=F_{y}\left(l_{1}+l_{2}c\right)-F_{z}l_{2}s.$$ (23)

## Simulation

According to Eqs (8) and (23), the torque response due to the interaction between the probe and soft tissue is dependent on the soft silicon phantom’s stiffness *k*_*t*_, probe’s stiffness *r*_*a*_, and indentation level *i*. Here we explore how different probing conditions such as: probe’s joint stiffness, indentation, and PSV would affect the distribution of torque response at the probe’s base during interaction with different phantom stiffness levels.

The expected value of the stiffness of the phantom, $k_{to}^{m}$, is identified to be 65 N/m with a standard deviation of 13.2 N/m. The source of uncertainty in the simulation is limited to that from the phantom’s stiffness. Based on the previous studies [1, 21], we can approximate the variability of the phantom’s stiffness to be a Gaussian distribution, $k_{t}∼N\left(k_{t}^{m},{k_{t}^{s}}^{2}\right)$, with expected value, $k_{t}^{m}$, and standard deviation, $k_{t}^{s}$. The changes in phantom’s stiffness, $\Delta k_{t}^{m}=k_{tn}^{m}-k_{to}^{m}$ = 10, 20, 30, and 40 N/m, represent the presence of a hard nodule at different depths respectively. The length of the nodule can be viewed as the contact duration with the probe; hence the longer this is, the slower the PSV (*v*_*probe*_). In the simulation, *v*_*probe*_ is classified in three levels, namely: slow (10 mm/s), medium (20 mm/s), fast (30 mm/s).

The models of tissue’s stiffness, in which the nodule is present, are given by

$$k_{t}^{m}=\left\{\begin{array}{cc} k_{to}^{m} & \text{if}\;0\;\leq \;t\;<\;t_{i}\;\text{and}\;t\;\geq \;t_{f}\\ k_{to}^{m}+\Delta k_{t}^{m}\sin\frac{\pi (t-t_{i})}{t_{f}-t_{i}} & \text{if}\;t_{i}\;\leq \;t\;<\;t_{f} \end{array}\right.$$

where

$$t_{i}=\frac{1}{2}\frac{L_{t}}{v_{probe}}\;\text{and}\;t_{f}=t_{i}+\frac{l_{n}}{v_{probe}}.$$ (24)

*L*_*t*_ = 200 mm, *l*_*n*_ = 15 mm, and *t* represent the length of the simulated phantom along the probing path, the diameter of the simulated nodule, and the simulation time respectively. *t*_*i*_ and *t*_*f*_ denote the time at which the probe’s tip first contacts and leaves the nodule’s area on the phantom’s surface respectively. We simulated the dynamic torque response, *τ*_*f*_, during interaction between the probe and the phantom under different conditions specified in Table 2. The simulations were carried out using ‘ode45’ in MATLAB R2013b, The Mathworks, Inc.

**Table 2:** Simulation parameters.

System	Variables	Value
**Probe**	*l*_1,2_	80, 70 [mm]
	*a*_1,2_	40, 35 [mm]
	*m*_1,2_	0.2, 0.3 [kg]
	*b*_*s*_	0.02 [Ns/m]
	*i*	{3, 5, 7, 9, 11} [mm]
	*v*_*probe*_	{10, 20, 30} [mm/s]
**Joint Stiffness**	*R*	6.8 [mm]
	*r*_*a*_	{0, 4, 8, 12, 16} [mm]
	*k*_*s*_	0.24 [N/mm]
**Silicon Phantom**	*ρ*	290 [mm]
	$k_{tn}^{m}$	{75, 85, 95, 105} [N/m]
	$k_{to}^{m}$	65 [N/m]
	$k_{tn,to}^{s}$	13.2 [N/m]
	*b*_*tn*,*to*_	0.1[Ns/m]

**Fig 5:**
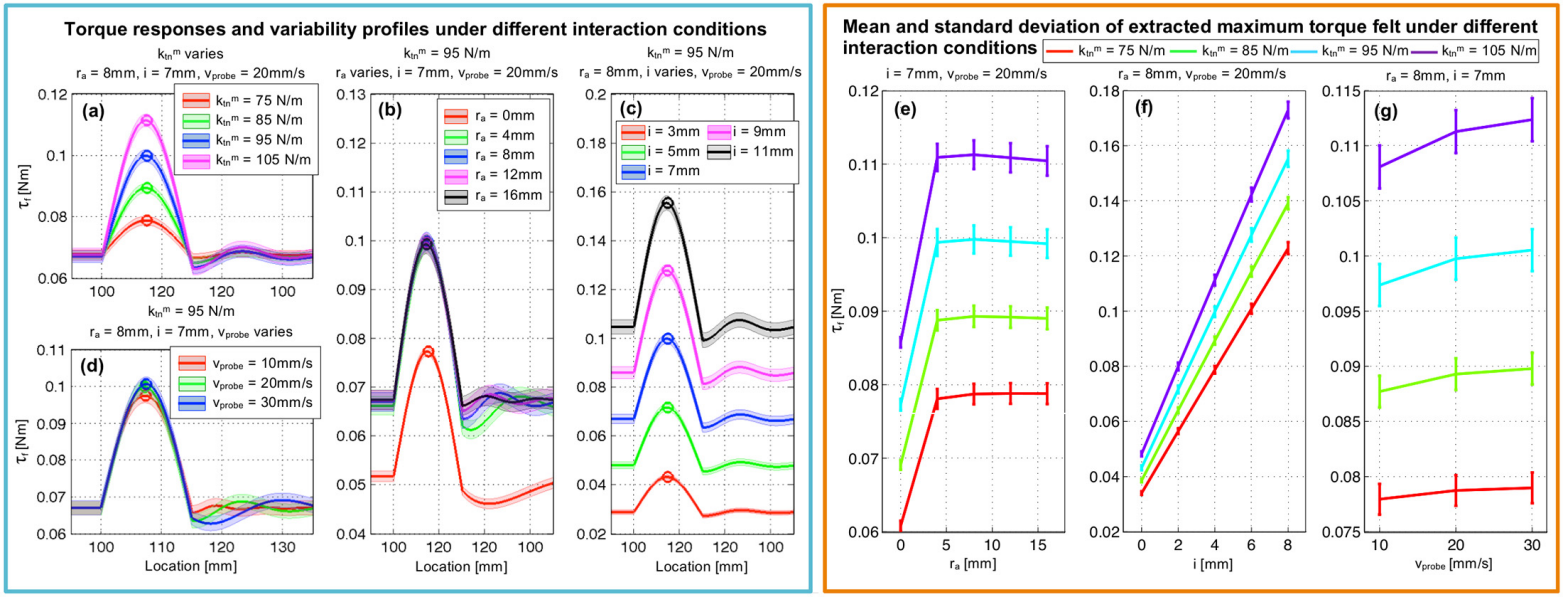
Sample of the simulated torque responses and the corresponding variability given different simulated interaction conditions across 25 simulation trials. The simulated spherical hard nodule of size 15 mm diameter is presented at location between 100 mm to 115 mm. The simulated interaction conditions for each sub-figure are as follows: (a) The average stiffness of soft silicon phantom is varied with the other parameters fixed at *r*_*a*_ = 8mm, *i* = 4mm, and *v*_*probe*_ = 20mm/s. (b) Average Stiffness of the probe is varied keeping the other variables fixed at $k_{tn}^{m}$ = 95N/m, *i* = 4mm, and *v*_*probe*_ = 20mm/s. (c) The indentation level of the probe, *i*, is varied keeping the rest parameters fixed at $k_{tn}^{m}$ = 95N/m, *r*_*a*_ = 8mm, and *v*_*probe*_ = 20mm/s. (d) The probing speed, *v*_*probe*_, is varied and the other parameters are kept at $k_{tn}^{m}$ = 95N/m, *r*_*a*_ = 8mm, and *i* = 4mm. For each torque profile measured during palpation, the maximum torque at the location of simulated hard nodule is extracted. Sub figures (e), (f), and (g) show the average maximum torque felt with error bars, under different combinations of probe’s internal stiffness, indentation, and PSV.

The sample of torque responses, *τ*_*f*_, and the variability resulted from the variability presented in the stiffness of the soft silicon phantom undergoing different interaction conditions across 25 simulation trials are shown in Fig 5(a)–5(d). Fig 5(a) represents the torque responses for different soft silicon phantom’s stiffness, $k_{tn}^{m}=\left\{75,85,95,105\right\}$ N/m; whereas the probe’s internal stiffness, indentation level, and PSV are fixed. This shows the monotonic increase in torque response as the stiffness of the phantom increases. Fig 5(b) to 5(d) represent the torque responses for different combinations of probe’s internal stiffness, indentation level, and PSV. As can be seen in Fig 5(b), *τ*_*f*_ increases as the internal stiffness of the probe (controlled by the position of anchor ring, *r*_*a*_) increases from *r*_*a*_ = 0 to 4 mm. After that, *τ*_*f*_ tends to settle down. The increase of the probe’s indentation level also elevates the torque responses as shown in Fig 5(c). In Fig 5(d), the influence of the PSV, *v*_*probe*_, on the torque response, given the fixed values for the rest of the simulation parameters, cannot be visually assessed. Therefore, we have applied a statistical method to determine this. Since the simulated torque response is normally distributed (this was tested using Kolmogrov-Smirnov test for normality), we can implement ANOVA (Analysis of Variance) test. The result from the test signifies no statistically significant difference between these torque response distributions (*p*-value > 0.05). Therefore, the change in PSV, *v*_*probe*_ does not significantly affect the torque response.

From each torque response profile measured during palpation, we extracted the maximum torque at the location of simulated hard nodule as shown in ‘circle’ in Fig 5(a)–5(d). Fig 5(e)–5(g) depict the expected values and standard deviations of extracted maximum torque response given different combinations of probe’s internal stiffness, indentation level, and PSV across 25 trials for $k_{tn}^{m}=$ {75(red line), 85(green line), 95(blue line), and 105(purple line)} N/m. Fig 5(e) presents the average maximum torque with standard deviation extracted from 25 simulation trials across different probe’s internal stiffness levels presented by *r*_*a*_. The average torque response increases as *r*_*a*_ increases from 0 to 4mm. Then the average peak torque response settles down. Furthermore, we see non-linear elevation of the torque standard deviation as the probe’s stiffness level increases. On the other hand, the average peak torque and the corresponding standard deviation shown in Fig 5(f) respectively tend to have a rather linear trend. Lastly, in Fig 5(g) we do not see any statistically significant difference in the average and standard deviation of peak torque response resulted from the change in the PSV.

These simulation results predict that the torque felt at the base of the probe can be controlled using probe stiffness, indentation level and PSV during the interaction with a soft tissue. The relationship between the torque felt and the combinations of probe’s stiffness, indentation level, and PSV are non-linear. Furthermore, in reality the variability present in the system is non-deterministic and may arise from several sources such as the probing behavior, environment, and the probe itself. These raise the question as to how we can exploit these non-linear relationships to enhance the interpretation of the features in the environment using proprioceptive feedback from the torque sensor mounted at the base of the probe (representing how the tendon sensor is located in natural muscles). Since the relationship is stochastic and non-linear, the best way to preserve the interaction information is to present the relationship in the form of a probabilistic distribution. It provides us the opportunity to implement an appropriate stochastic machine learning technique to understand the role of individual factor in the interpretation of haptic perception.

## Experiments and Results

In the experiment, we use the controllable stiffness robotic probe described earlier to derive deeper insights into the influence of the variation of combinations of probe’s internal stiffness, indentation level, and PSV on the real-time estimation of the depth of a hard nodule embedded in soft silicon phantom. We explore whether a probe with controllable stiffness, indentation level, and PSV can exploit its past experience of palpation by varying its own internal stiffness, indentation level, and PSV to estimate the depth of embedded nodule inside a soft silicon phantom.

### Palpation Memory Primitives

The palpation experiences during interaction with the soft silicon phantoms with a nodule embedded at different depths can be presented in the form of a probabilistic representation, which hereafter is referred to as ‘memory primitives’. The memory primitives were built from torque measurements, *τ*_*f*_, for different *r*_*a*_, *v*_*probe*_ and *i*, over multiple palpation learning trials.

The probe mounted under the XY-table was programmed to palpate in a straight line along the probing path over the soft silicon phantom’s exposed surface shown in Fig 2(d). During each palpation trial, the torque response due to the interaction is measured at the base around the x-axis at 1000 Hz. Readings of *r*_*a*_, *i*, *v*_*probe*_, and *τ*_*f*_ were recorded using LabVIEW2012 software, National Instruments Corp, through the data acquisition cards. Data processing was carried out using MatLAB R2013b, The MathWorks, Inc.

In order to construct the primitives for this experiment, we conducted palpation experiments across 5 probe stiffness levels, *r*_*a*_, 5 indentation levels, *i*, 3 levels of PSV, *v*_*probe*_, and 3 levels of nodule depths, *d*. The experimental conditions are shown in Table 3. These amount to 225 unique interaction conditions. For each given combination, 25 palpation trials were repeated to allow the formation of distribution of peak torque response.

**Table 3:** Experimental Conditions.

Experimental variables	Sym.	Values	Units
Probe’s stiffness (anchor position)	*r*_*a*_	{0,4,8,12,16}	mm
Relative distance between the tip of the probe at rest and the surface of phantom, i.e. inwards the phantom (indentation)	*i*	{3,5,7,9,11}	mm
Probe’s velocity	*v*_*probe*_	{10,20,30}	mm/s
Nodule’s depth	*d*	{2,4,8}	mm
Distance between the XYplate and bottom of phantom	*l*_*t*_	320	mm

**Fig 6:**
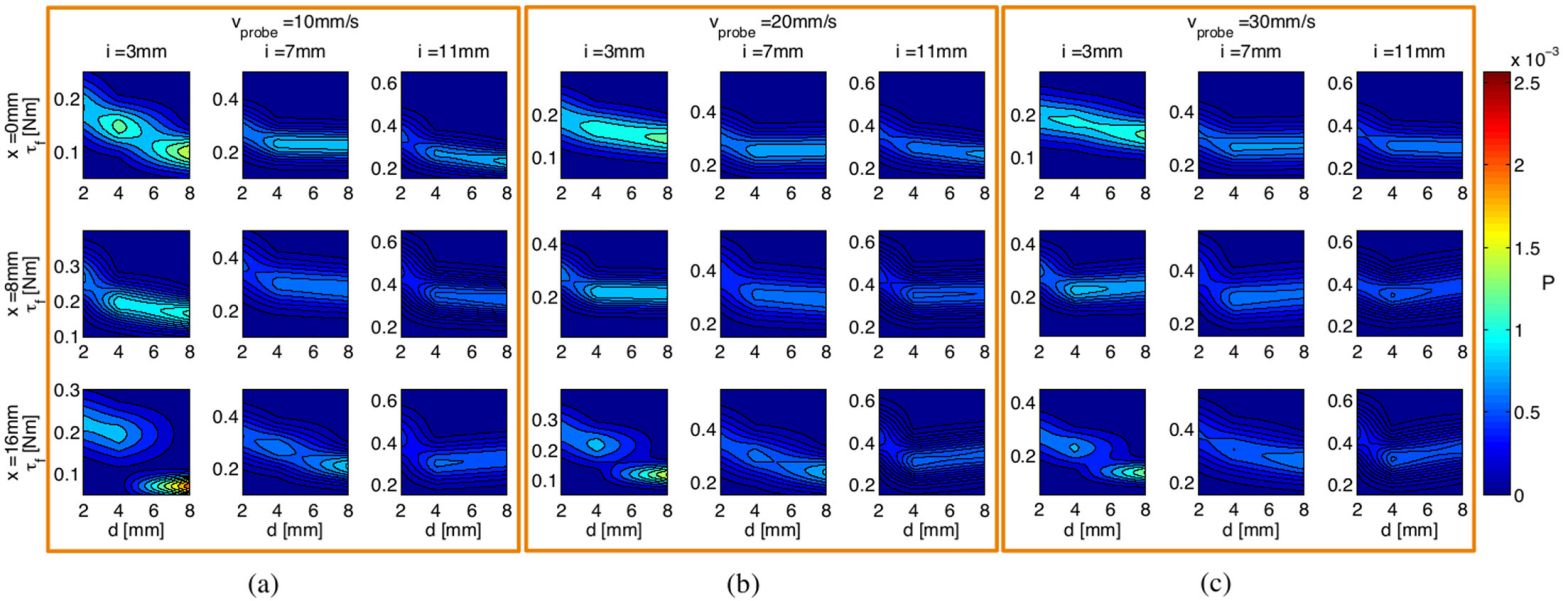
Examples of memory primitives computed as probability functions of the de-noised torque profiles from 25 trials given different interaction conditions shown in Table 3. The sample of memory primitives shown here consist of those when the PSV, *v*_*probe*_ = 10, 20, and 30, in subfigures (a), (b), and (c), for the indentation level, *i*, of 3, 7, and 11 mm, and the stiffness of the joint denoted by *r*_*a*_, of 0, 8, and 16 mm.

Each measured torque signal from the F/T transducer is de-noised for 5 levels using wavelet decomposition technique with a Daubechie’s *db*10 mother wavelet. The peak torque at the nodule’s location is then extracted from each de-noised signal. The peak torque distribution given different combinations of the probe’s stiffness, indentation level, and the probing speed for different nodule’s depth level, *P*(*τ*_*f*_|*d*, *r*_*a*_, *i*, *v*_*probe*_), can be constructed from all 25 trials by fitting a normal distribution to the data. Here, only 81 interaction conditions are depicted as examples of memory primitives as shown in Fig 6(a), 6(b) and 6(c).

### Experimental results and analysis

The non-linear relationship between the measured torque probability distribution and different combinations of probe’s stiffness, indentation level, and PSV can be used in an appropriate machine learning algorithms to enhance the accuracy in nodule’s depth estimation. In this paper, interpretation of real-time torque measurements during palpation was done using memory primitives (conditional probability density functions) in a Bayesian Inferencing framework as given by

$$P_{t}\left(d|\tau_{f}\right)=\frac{P\left(\tau_{f}|d,r_{a},i,v_{probe}\right)P_{t-1}\left(d\right)}{\sum_{n=1}^{m}P\left(\tau_{f}|d_{n},r_{a},i,v_{probe}\right)P_{t-1}\left(d_{n}\right)},$$ (25)

where *P*_*t*_(*d*|*τ*_*f*_) is the posterior probability of nodule’s depth given *τ*_*f*_. *t* denotes the current estimation iteration. *P*_*t*−1_(*d*) represents the prior distribution of *d*. *P*(*τ*_*f*_|*d*, *r*_*a*_, *i*, *v*_*probe*_) refers to the likelihood probability distribution of torque, given *d*, *r*_*a*_, *i*, and *v*_*probe*_. *n* indicates the index of *d*. And *m* = 3 is the number of possible classes for nodule depth.

#### Bayesian Inference in Estimating the Nodule’s depth

Table 3 shows different combinations of probe-soft tissue interaction conditions used to assess the performance of the Bayesian Inferencing algorithm 1 to estimate *d* across 5 iterations. The assessment of the nodule’s depth estimation was repeated for 10 trials in order to obtain the average accuracy of estimation. In each assessment trial, the memory primitives given different combinations of probe’s interacting conditions were constructed from 25 randomly chosen learning trials.

**Algorithm 1:** Nodule’s depth estimation algorithm using Bayesian Inference

**1** function DepthEstimation (*τ*_*f*_*t* = 1..5__(*d*_*r*_, *r*_*a*_, *i*, *v*_*probe*_));

 **Input:** Real time torque reading, *τ*_*f*_*t* = 1..5__(*d*_*r*_, *r*_*a*_, *i*, *v*_*probe*_)

 **Output:** Depth estimation accuracy

**2** Define *P*_*t* = 0_(*d*) as a flat distribution across different *d*;

**3** **for** *each combination of* {*r*_*a*_, *i*, *v*_*probe*_}, *and actual nodule’s depth*, *d*_*r*_ **do**

**4** **for** *each iteration* *t* ∈ 1..5 **do**

**5**  Retrieve and process new *τ*_*f*_*t*__ from the sensor reading, given known probing bahavior {*r*_*a*_, *i*, *v*_*probe*_}.;

**6**  Compute *P*(*τ*_*f*_*t*__|*d*, *r*_*a*_, *i*, *v*_*probe*_) from ***φ***.;

**7**  Recall prior distribution of hypothesis of nodule’s depth *P*_*t*−1_(*d*).;

**8**  Compute *P*_*t*_(*d*|*τ*_*f*_) using Eq 25.;

**9**  Store posterior distribution as a prior distribution for the next iteration.;

**10**  $d_{est}=\underset{m}{\mathrm{argmax}}\left(P_{t}\left(d|\tau_{f}\right)\right)$ ;

**11** **end**

**12** **end**

**Fig 7:**
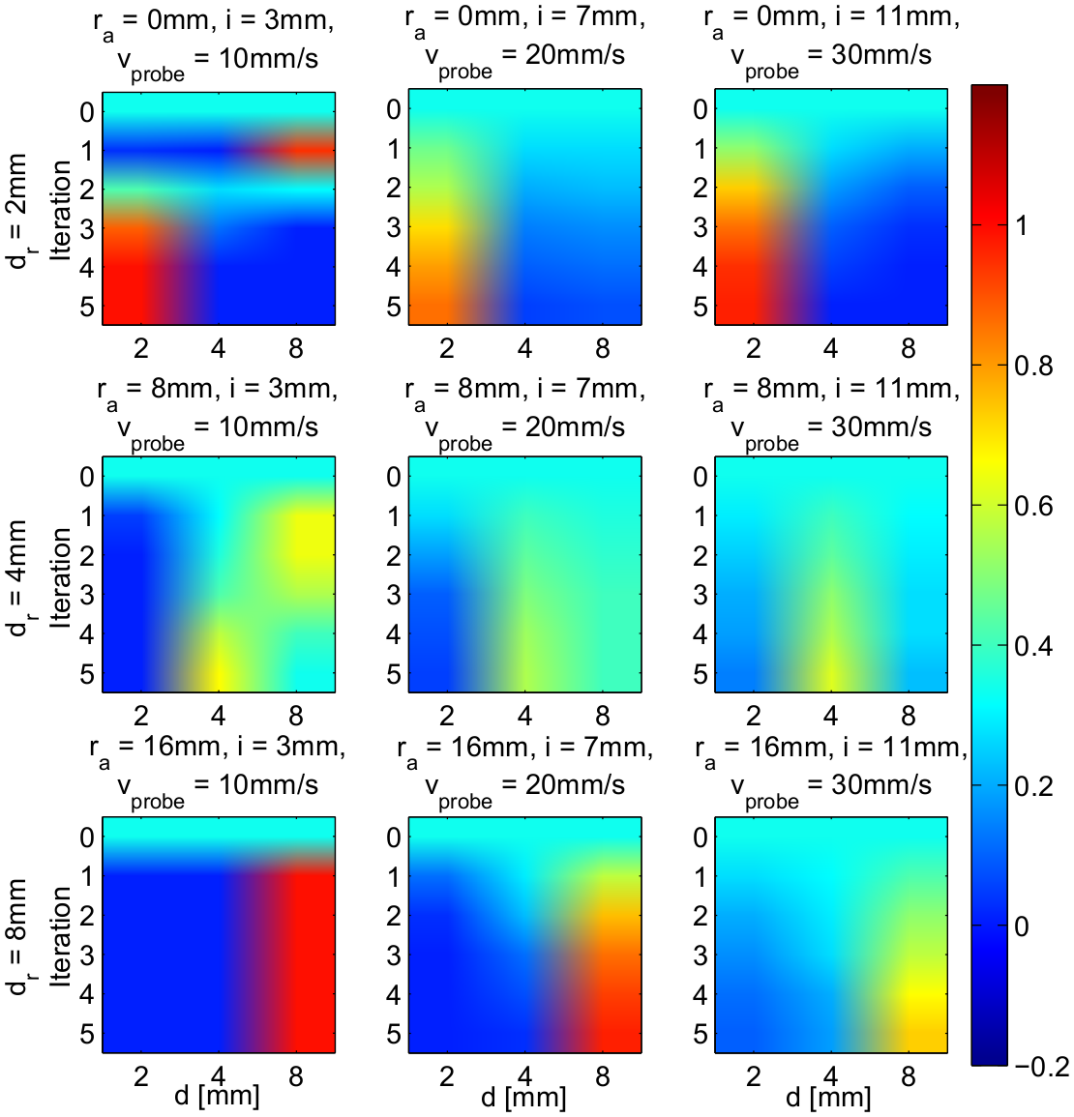
Examples of posterior distribution of nodule’s depth estimation across iterations. Each plot depicts the distribution of depth estimation from the initially defined flat distribution at *t* = 0 to *t* = 5 given different combinations of probe’s stiffness, indentation, and PSV. The real depths of nodule, *d*_*r*_, assessed here were those known values associated with the memory primitives explained earlier.

Fig 7 shows an example of a nodule depth estimation trial that consists of 5 iterations of Bayesian Inference for a given combination of probe’s stiffness, indentation, and PSV. Each subplot shows the progression of the posterior probability of nodule’s depth estimation starting from a flat distribution at *t* = 0 to a refined one at *t* = 5.

**Fig 8:**
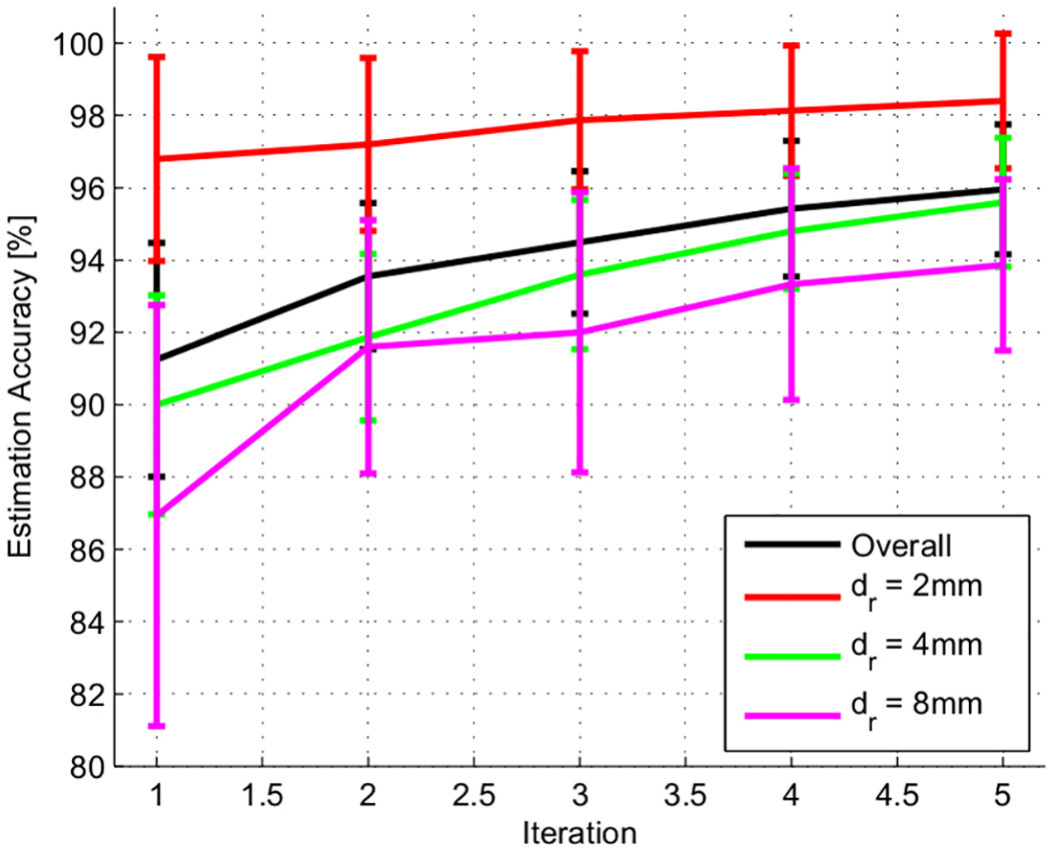
The nodule’s depth estimation accuracy under fixed-5-iteration Bayesian inferencing algorithm. The resulting overall nodule’s depth estimation accuracy is shown in black line. The estimation accuracy for each actual depth, *d*_*r*_ = 2, 4, and 8 mm are shown in red, green and magenta lines respectively.

The overall accuracy across 10 assessment trials in nodule depth estimation after each iteration and those for different nodule depth levels are shown in Fig 8. On average, the overall nodule depth estimation accuracy increases from approximately 91% with standard deviation of 3.23% at the first iteration (*t* = 1) to 96% with standard deviation of 1.8% at (*t* = 5). We witness that the estimation accuracy of nodule depth decreases as the nodule is buried deeper from the exposed surface from 99.3% at *d* = 2 mm to 95.2% and 94.2% at *d* = 4 and 8 mm, respectively. Higher iteration numbers cause the expected values of estimation accuracy for all depth ranges to increase and the standard deviations to decrease.

Further statistical analysis was performed to investigate the significance of *r*_*a*_, *i*, and *v*_*probe*_, on the depth estimation accuracy across all assessment trials. The Kolmogrov-Smirnov test showed that the nodule’s depth estimation was not normally distributed *p* > 0.05. Hence, the conventional Analysis of Variance (ANOVA) could not be performed. Therefore, a non-parametric Kruskal-Wallis method was applied. The resulting *p*-values from the test were 0.0002, 0.4715, and 0.7394 for probe’s stiffness, *r*_*a*_, indentation, *i*, and PSV, *v*_*probe*_, respectively over the course of 10 assessment trials across different combinations. Therefore, probe’s stiffness, *r*_*a*_, has statistically significant contribution towards the nodule’s depth estimation accuracy (*p*-value <0.05); while the variation of *i* and *v*_*probe*_ do not have a significant influence.

The average accuracy in the estimation of nodule’s depth given different probe’s stiffness, *r*_*a*_, indentation level, *i*, and PSV, *v*_*probe*_ across 10 assessment trials are shown in Tables 4, 5 and 6, respectively. We can see that the average nodule’s depth estimation is slightly less accurate for the case when the joint of the probe is both stiffest and most relaxed; while it is most accurate when *r*_*a*_ = 12 mm. This suggests that the regulation of body’s stiffness matters when making an estimation about the environment. Nonetheless, it is important to notice that the efficacy of *r*_*a*_ = 12 mm varies depending on the probing speed and the indentation used.

**Table 4:** Nodule’s depth estimation accuracy across *r*_*a*_.

	*r*_*a*_ [mm] (Probe’s stiffness)				
*d*_*r*_ [mm]	0	4	8	12	16
**2**	96.7%	98.7%	98.7%	99.3%	98.7%
**4**	90.7%	97.3%	93.3%	98.7%	98%
**8**	95.3%	83.3%	92.7%	100%	98%
**Overall**	94.2%	93.1%	94.9%	99.3%	98.2%

**Table 5:** Nodule’s depth estimation accuracy across *i*.

	*i* [mm] (Indentation)				
*d*_*r*_ [mm]	3	5	7	9	11
**2**	94.7%	100%	100%	99.3%	98%
**4**	96%	94.7%	90%	98.7%	98.7%
**8**	89.3%	96%	90%	98.7%	95.3%
**Overall**	93.3%	96.9%	93.3%	98.9%	97.3%

**Table 6:** Nodule’s depth estimation accuracy across *v*_*probe*_.

	*v*_*probe*_ [mm/s] (probing vel.)		
*d*_*r*_ [mm]	10	20	30
**2**	97.6%	100%	97.6%
**4**	96.4%	94.4%	96%
**8**	88%	96%	97.6%
**Overall**	94%	96.8%	97.1%

Furthermore, Fig 7 suggests that the posterior distribution of nodule’s depth estimation can converge at different rates depending not only on the combination of *r*_*a*_, *i*, and *v*_*probe*_, but also on the noise level of real-time sensor measurements in each iteration and the chosen memory primitives in the likelihood function. This leads to the question as to whether we can determine the sufficiency of the number of iteration/exploration required to make an accurate estimation of the nodule depth. We can address this question by computing the information transfer entropy in each iteration. This will be addressed in detail in the next section.

#### Kullback-Liebler Transfer Entropy

In general, the common influences of multiple coupled systems and factors can be quantified through the directed information exchanges by measuring the information transfer entropy, also known as relative entropy [22]. For example, we can assign the combination of internal stiffness, indentation level, and PSV; and the torque sensor reading to be random variables (RV-A) and (RV-B) respectively. While the mutual information of two coupled variables between RV-A and RV-B does not change with the exchanges of variables; the transfer entropy from RV-A to RV-B is not identical to that from RV-B to RV-A. Transfer entropy can be quantified using Kullback-Liebler (KL) divergence.

To be more specific, KL-divergence can be used to assess whether further information regarding the nodule’s depth estimation can be gained by taking another action (further iteration in Bayesian nodule’s depth estimation procedure). If we consider a set of *P*_*t*_(*d*|*τ*_*f*_) computed at the end of each Bayesian iteration as the hypothesis of the depth estimation, its entropy for a given torque measurement, *τ*_*f*_, is dependent on a set of probe’s stiffness, indentation level, and PSV, {*r*_*a*_, *i*, and *v*_*probe*_}. KL-divergence defined in Eq (26) represents the additional information gained, *G*, about the relationship between the hypothesis of depth estimation, *P*_*t*_(*d*), and *τ*_*f*_ across iterations of Bayesian Inference as well as across different sets the probe’s stiffness, indentation level, and PSV. Therefore, KL-divergence is a good measure to quantify the gain of different actions underlying the changes in the behavior.

$$G_{t}=P_{t}\left(d|\tau_{f}\right)\log\frac{P_{t}\left(d|\tau_{f}\right)}{P_{t=0}\left(d\right)},$$ (26)

*P*_*t*_(*d*|*τ*_*f*_) represents the probability distribution of depth estimation which is obtained from the Bayesian inference shown in Eq (25) at *t*^*th*^ iteration, and *P*_*t* = 0_(*d*) represents the base hypothesis about the nodule’s depth estimation.

#### Bayesian Inference in Estimating the Nodule’s depth with Kullback-Liebler Divergence

In addition to the Bayesian Inference method for estimating the depth of the nodule from the real-time captured torque data, here KL-divergence is implemented at the end of each Bayesian iteration to determine whether further measurement is required to accurately estimate the nodule’s depth. This additional process is carried out by computing the correlation distance, *δ*, between information gain from the current hypothesis, *G*_*t*_, and that from the prior hypothesis, *G*_*t*−1_, in relation to the base prior distribution, *P*_*t* = 0_(*d*). No further measurement is necessary to make an accurate estimation when the correlation distance, *δ*, is less than the empirically specified threshold of *T* = 0.0005. This signifies that there is negligible change in the information gained across the iterations and the distribution of the nodule’s depth estimation hypothesis has converged. The depth estimation procedure is shown in Algorithm 2. Similar to the process presented in previous section, the assessment of the nodule’s depth estimation is repeated for 10 assessment trials to obtain the average accuracy in the estimation. In each assessment trial, the memory primitives given different combinations of probe’s interacting conditions are constructed from 25 randomly chosen learning trials.

**Algorithm 2:** Nodule’s depth estimation algorithm using Bayesian Inference and KL divergence

**1** function DepthEstimationKL(*τ*_*f*_(*d*_*r*_, *r*_*a*_, *i*, *v*_*probe*_));

 **Input:** Real time torque reading, *τ*_*f*_(*d*_*r*_, *r*_*a*_, *i*, *v*_*probe*_)

 **Output:** Depth estimation accuracy

**2** Define *P*_*t* = 0_(*d*) as a flat distribution across different *d*;

**3** **for** *each combination of* {*r*_*a*_, *i*, *v*_*probe*_}, *and actual nodule’s depth*, *d*_*r*_ **do**

**4** *δ* = 1 Initialize correlation distance to 1;

**5** *t* = 0 Initialize the number of iteration to 0;

**6** *G*_0_ = 0; Initialize the information gain at *t* = 0 to 0;

**7** **while** *δ* > *T* **do**

**8**  *t* = *t* + 1;

**9**  Follow Step 5–9 in Algorithm 1;

**10**  Compute *G*_*t*_ using Eq (26);

**11**  Compute *δ* between *G*_*t*_ and *G*_*t*−1_.;

**12** **end**

**13** $d_{est}=\underset{m}{\mathrm{argmax}}\left(P_{t}\left(d|\tau_{f}\right)\right)$;

**14** **end**

**15** Compute the nodule’s depth estimation accuracy.

**Fig 9:**
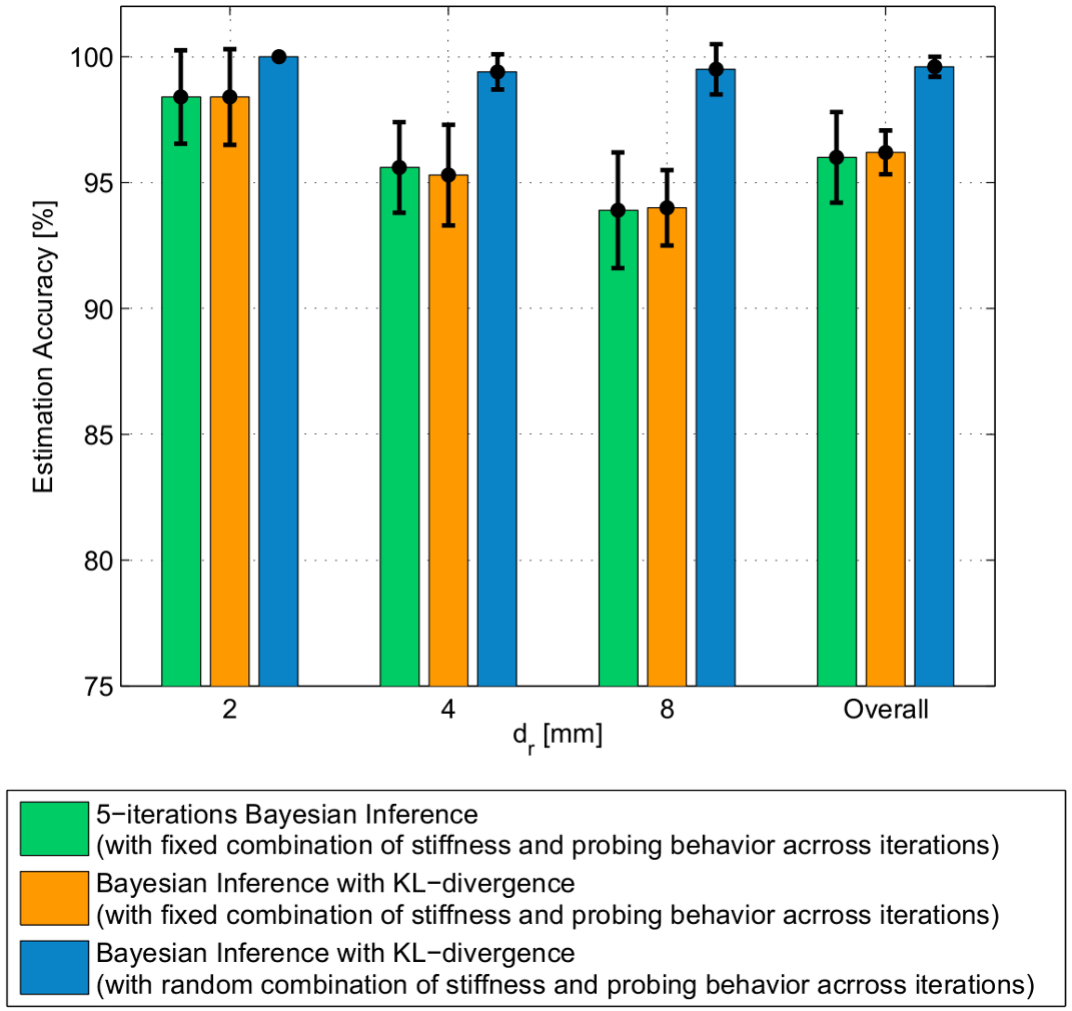
Overall nodule’s depth estimation accuracy when using different approaches. 1) 5-iteration Bayesian inference without KL-divergence (shown in green), 2) the Bayesian Inference together with the KL-Transfer Entropy with fixed probe’s stiffness, indentation level, and PSV (shown in orange), and 3) the Bayesian Inference together with the KL-Transfer Entropy with random probe’s stiffness, indentation level, and PSV (shown in blue).

With the implementation of KL-divergence in addition to the Bayesian Inference algorithm, the nodule’s depth estimation process requires on average of only 2.8 iterations with standard deviation of 1.2 iterations to converge. While the number of iterations required for convergence is kept to minimum; the nodule’s depth estimation accuracy still reaches within the comparable range to that with fixed 5-iterations in the inferencing algorithm presented in Algorithm 1. On average the overall depth estimation accuracy is approximately 96.2% as shown in Fig 9 (orange bars). The accuracy of nodule’s depth estimation for each actual depth are approximately 98.4%, 95.3%, and 94% for *d*_*r*_ = 2, 4, and 8 mm respectively. These results show that this method minimizes the number of explorations needed to make an accurate estimation about the depth of the nodule.

Therefore, we can conclude that Bayesian Inference together with KL-divergence provides a real-time framework to estimate the convergence to an optimal estimate of nodule depth in the sense of information gain.

#### Bayesian Inference in Estimating the Nodule’s depth with Dynamic Probing

So far, the experiments involved keeping *r*_*a*_, *i*, and *v*_*probe*_ constant given a set of probing iterations in a trial. However, biological counterparts like humans regulate the internal impedance more like a random variable within a given probing attempt. Therefore, we conducted further experiments to explore whether we can enhance the nodule’s depth estimation accuracy by allowing changes in the combination of probe’s stiffness, indentation level, and PSV across trials. This allows the estimation process to explore in multiple search spaces (memory primitives).

**Algorithm 3:** Nodule’s depth estimation algorithm using Bayesian Inference and KL divergence

**1** function DepthEstimationKLR(*τ*_*f*_(*d*_*r*_, *r*_*a*_, *i*, *v*_*probe*_));

 **Input:** Real time torque reading, *τ*_*f*_(*d*_*r*_, *r*_*a*_, *i*, *v*_*probe*_)

 **Output:** Depth estimation accuracy

**2** Define *P*_*t* = 0_(*d*) as a flat distribution across different *d*;

**3** **for** *each actual nodule’s depth*, *d*_*r*_ **do**

**4** Follow Step 4–6 in Algorithm 2;

**5** **while** *δ* > *T* **do**

**6**  *t* = *t* + 1;

**7**  Randomly select combination of {*r*_*a*_, *i*, and *v*_*probe*_};

**8**  Follow Step 9–11 in Algorithm 2;

**9** **end**

**10** $d_{est}=\underset{m}{\mathrm{argmax}}\left(P_{t}\left(d|\tau_{f}\right)\right)$ ;

**11** **end**

**12** Compute the nodule’s depth estimation accuracy.

In order to address this, we repeated a similar estimation algorithm to that shown in Algorithm 2. However, instead of the static combination of probe’s stiffness, indentation level, and palpation velocity; these variables were allowed to randomly vary across iterations in the nodule’s depth estimation process. We repeated this process for 100 trials for each artificial soft silicon phantom with nodule embedded at *d*_*r*_ = 2, 4, and 8 mm. The estimation procedure is shown in Algorithm 3. At the beginning of the estimation procedure, the process randomly selects the probe’s stiffness, indentation level, and PSV combination. In each iteration, this combination is arbitrarily varied to allow the exploration in the other memory primitives to inference the previous posterior.

The nodule’s depth estimation result from the implementation of the Bayesian Inference with dynamic probing shown in Algorithm 3 are shown in blue bar in Fig 9. The overall average accuracy from 100 trials using this algorithm as the estimation hypothesis converges reaches 99% with standard deviation of 0.5%. For each individual depth level, the average estimation accuracy were all significantly higher; while the corresponding standard deviations were lower compared to those from the Bayesian Inference with static set of probe’s stiffness, indentation level, and PSV combinations. The result from this assessment also confirms our initial hypothesis that the average number of iterations required to perform accurate depth estimation is kept to a minimum at approximately 3 iterations with standard deviation of 1.3 iterations.

**Fig 10:**
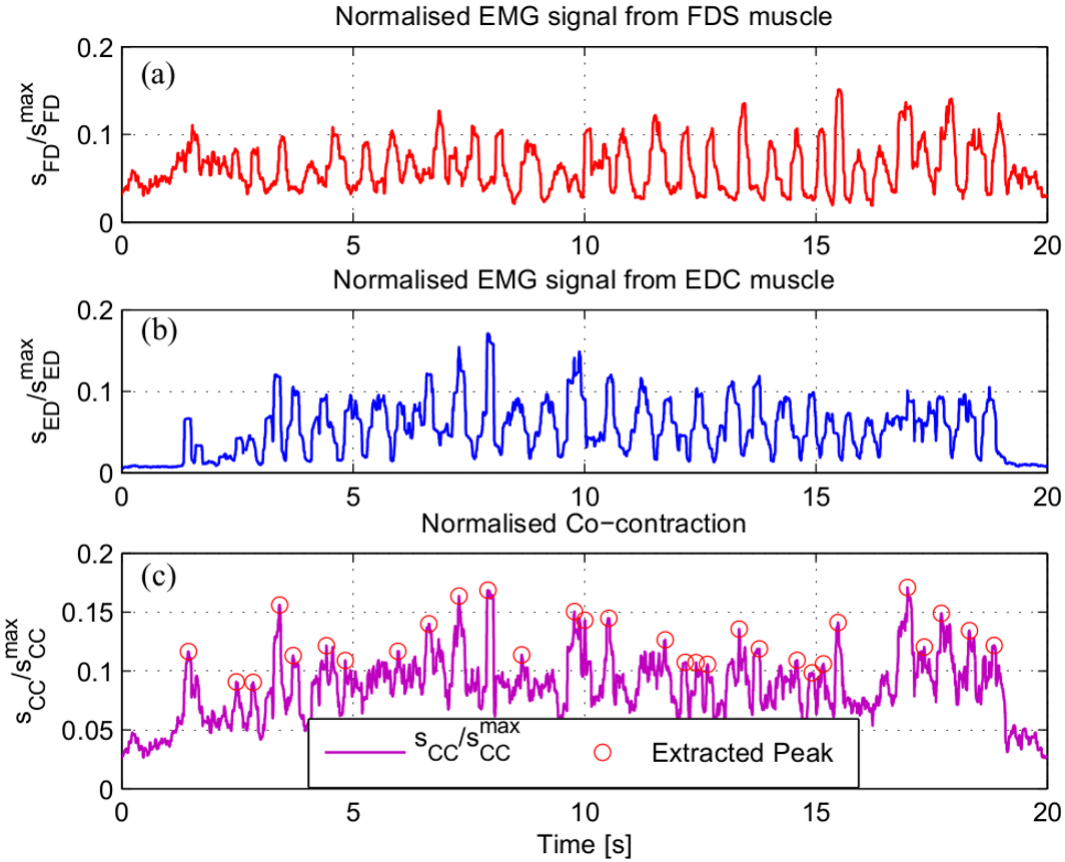
Sample of muscle activity at FDS and EDC during human’s manual palpation. The FDS (a) and EDC (b) muscle activities were quantified by the EMG signal during manual palpation trial to estimate the depth of a hard nodule embedded inside a silicon phantom. The combination of the activities contributed from both muscles can be described as the co-contraction. The normalised co-contraction is shown in magenta curve in (c); whereas the red circles indicate the peaks extracted from this signal.

A preliminary experiment was carried out with 1 human subject in the same probing task. In order to have comparative basis between human and robotic experiments, the subject was blindfolded and asked to palpate the same set of soft phantoms to estimate the depth of the embedded hard nodule. The muscle activity caused by the stiffness regulation of the finger was captured using electromyography (EMG) signal at Flexor digitorum superficialis (FDS) and Extensor digitorum communis (EDC) [23]. The example of both normalised EMG signals are shown in Fig 10(a) and 10(b). The combination of both EMG signals results in the co-contraction activity of the muscle pair. The muscle co-contraction pattern of a human subject during manual palpation is shown in Fig 10(c). The peaks (circled in red in Fig 10(c)) indicate the co-activation of the muscle pair, when both FDS and EDC are contracted. Similar to the robotic experiment with the implementation of Algorithm 3, the human’s co-contraction pattern also demonstrates variability. The variability of both EMG signals arises from the changes in the finger’s stiffness level of which the activation of FDS and EDC muscle pair are responsible for. The regulation of internal stiffness level of the human’s finger can be correlated with the change in the joint’s stiffness of the robotic probe. Our results based on robotic experiments explain the reason behind this activity.

## Discussion and Conclusion

This paper investigated both individual and collective role of the probe’s internal stiffness, indentation level, and PSV in the accuracy in interpreting and estimating an environmental feature (depth of a nodule) by controlling a soft probe. The soft robotic probe comprised of a variable stiffness joint and an indentation level control mechanism. The probe structure was mounted under an XY-stage allowing the planar movement. Firstly, we simulated the dynamics of palpation using the designed probe on a simulated soft silicon phantom to observe the interaction between them under different combinations of probe’s internal stiffness, indentation level, and PSV.

The results from the simulation suggest that 1) the torque felt at the base of the probe can be controlled using different combinations of probe’s stiffness, indentation level, and probing speed and 2) the relationships between the torque measured, the stiffness of the soft silicon phantom, and the combination of probe’s internal stiffness, indentation level, and PSV are non-linear. While a variability in the simulated system from the Gaussian distribution of the phantom’s stiffness is pre-defined; the variability in such a system in reality is non-deterministic and can arise from multiple sources. These brought into a question as to how we can use these non-linear relationships in the experiment to enhance the estimation of the environmental features.

In the experiment, we investigated the question as to how the probe with controllable stiffness, indentation level, and PSV can exploit its past experience of palpation to estimate the depth of a nodule embedded inside a soft tissue in real time. The non-linear relationship between the probe’s measured torque, its internal variables, and the environment (depth of nodule in silicon phantom) were presented in the form of a probabilistic distribution given different combinations of probe’s internal stiffness, indentation level, and PSV. In this paper, we referred to these conditional probability distributions as ‘memory primitives’. These ‘memory primitives’ functioned as likelihood functions in a Bayesian framework to estimate the depth of a nodule in the soft tissue phantom. The memory primitives were constructed from three levels of PSV, five levels of indentation, and five levels of joint stiffness, for three nodule’s depth levels. In total 5625 probing trials were performed using this automated experimental setup.

In conclusion, the implementation of Bayesian Inference allows the algorithm to accurately estimate the depth of a nodule from the measured torque real-time. Furthermore, KL-divergence was introduced to determine whether further iteration of measurement is required to make an accurate estimation by comparing the information gained in the current iteration to that of the previous iteration. It was shown that on average the estimation processes using Algorithm 2 and 3 require approximately 3 iterations to converge in order to obtain comparable and better (in the latter) estimation accuracy. Finally, allowing the combination of probe’s internal stiffness, indentation level, and PSV to randomly vary across iterations (allowing exploration in multiple memory primitives in each nodule’s depth estimation process), resulted in a convergence to the global optimum with a minimum number of iterations. We showed that, this could enhance the average depth estimation accuracy to almost 100% with higher repeatability (smaller standard deviation).

The insights from this study sheds light on the practice of manual and robotic assisted palpation of soft tissue to locate T-1 stage tumors in biological tissues [18]. Medical literature shows that T-1 stage tumors can be modeled as spherical shape hard nodules. Since the focus of this paper is to understand the importance of the internal impedance of the probe in detecting a hard nodule in a soft tissue, we limited the study to a spherical acrylic hard nodule buried at depths upto 8mm. This scenario represents the conditions of a typical manual tumor localizing procedure for a T-1 stage tumor. Even within this range of depths, Fig 9 shows how the accuracy of nodule’s depth estimation decreases as the depth of the nodule increases. Nodules located deeper in the tissue would require higher indentation forces potentially causing damage to the tissue. However, future studies could be done with different shapes and materials of nodules buried deeper in the tissue.

This paper has provided important explanations about the role of morphological computation in haptic based probing of a soft object, as well as providing guidelines to design and control variable stiffness probes for physical examination. Certainly, the operational implementation of this probe should be further developed depending on different applications. However, the fact that controllable internal stiffness helps to gain proprioception information is still valid in such a tool. However, additional complexities arising from factors such as variable friction and irregular surface conditions not addressed in this paper should be further examined. Future studies will also involve temporal control of probe stiffness, indentation, and speed to better understand diverse probing strategies used by different classes of human participants as seen in [1].
